# Real-world use of tafasitamab for relapsed or refractory diffuse large B-cell lymphoma among racial and ethnic minorities in the United States

**DOI:** 10.1093/oncolo/oyag357

**Published:** 2026-09-12

**Authors:** Narendranath Epperla, Loretta J Nastoupil, Bruce Feinberg, John Galvin, Prathamesh Pathak, Theresa Amoloja, Danielle Gentile, Parisa Asgarisabet, Kim Saverno

**Affiliations:** Division of Hematology, The Ohio State University, Columbus, OH 43201, United States; Department of Lymphoma/Myeloma, The University of Texas MD Anderson Cancer Center, Houston, TX 77030, United States; Present address: Southwest Oncology, CommonSpirit Oncology Mercy, Durango, CO 81301, United States; Cardinal Health, Dublin, OH 43017, United States; Incyte Corporation, Wilmington, DE 19803, United States; Cardinal Health, Dublin, OH 43017, United States; Incyte Corporation, Wilmington, DE 19803, United States; Cardinal Health, Dublin, OH 43017, United States; Cardinal Health, Dublin, OH 43017, United States; Incyte Corporation, Wilmington, DE 19803, United States

**Keywords:** diffuse large B-cell lymphoma, ethnicity, immunotherapy, race

## Abstract

**Background:**

Outcomes may differ among patients with diffuse large B-cell lymphoma (DLBCL) based on race and ethnicity; however, individuals from diverse racial and ethnic backgrounds have historically been underrepresented in clinical trials. This study describes the real-world characteristics, treatment patterns, and outcomes of patients treated with tafasitamab, an anti-CD19 monoclonal antibody for relapsed or refractory (R/R) DLBCL, by race and ethnicity.

**Methods:**

A descriptive subgroup analysis by race and ethnicity was conducted from a retrospective, physician-abstracted medical chart review study. Patients were aged ≥18 years, initiated tafasitamab treatment for R/R DLBCL on/after October 21, 2020, and had ≥4 months follow-up; patients who died during follow-up were included. Data were collected from February 22 to March 29, 2023, and from December 18, 2023 to January 31, 2024.

**Results:**

The analysis included 181 patients: 64.1% White, 22.1% Black or African American, 7.7% Other (race other than White, Black or African American, or unknown), 6.1% were of unknown race; 82.3% non-Hispanic, 17.1% Hispanic, and 0.6% were of unknown ethnicity. The median (Q1-Q3) follow-up time for the overall study population from tafasitamab initiation was 14.7 (8.5-17.1) months and was relatively similar for all subgroups. Most patients (71.8%) received tafasitamab in second-line and this was true across subgroups. Real-world overall response rates (95% CI) were 73.3% (65.2-81.3), 82.5% (70.7-94.3), 74.5% (67.5-81.5), and 67.7% (51.3-84.2) for White, Black or African American, non-Hispanic, and Hispanic patients, respectively.

**Conclusions:**

Findings suggest effectiveness outcomes among patients receiving tafasitamab for R/R DLBCL were consistent across racial and ethnic groups.

Implications for practice This real-world study provides important evidence in our understanding of tafasitamab benefit among patients from racial and ethnic backgrounds typically underrepresented in clinical trials. Our analysis describes real-world characteristics and outcomes of patients with relapsed or refractory diffuse large B-cell lymphoma (R/R DLBCL) treated with tafasitamab when examined by race and ethnicity. Findings suggest that effectiveness outcomes in patients receiving tafasitamab for R/R DLBCL were consistent across various racial and ethnic groups.

## Introduction

Although cancer incidence rates are higher among certain racial and ethnic subgroups for many types of cancer, 5-year survival rates for all cancers combined continue to be higher among non-Hispanic White patients compared with other racial and ethnic groups, with lower survival rates observed for non-Hispanic Black, non-Hispanic American Indian or Alaska Native, and Hispanic patients.^1^ Among patients with the most common type of B-cell non-Hodgkin lymphoma (NHL), diffuse large B-cell lymphoma (DLBCL), treatment outcomes vary based on racial and ethnic identity.^2–4^

DLBCL accounts for approximately 1 in 3 new diagnoses of B-cell NHL in the United States.^5^ The Surveillance, Epidemiology, and End Results (SEER) database indicated that the age-adjusted rate of new cases per 100 000 persons per year between 2018 and 2022 was higher among Hispanic patients (7.3 for Hispanic males and 5.6 for Hispanic females) than non-Hispanic White patients (6.7 for male and 4.5 for female) and non-Hispanic Black patients (5.0 for male and 3.3 for female).^6^ Population-based analyses of patients with DLBCL, including retrospective studies of the SEER database registries, show significant differences in outcomes based on race and ethnicity; minority racial groups have lower 5-year and overall survival rates and increased cancer mortality compared with White patients.^2^^,^^7^^,^^8^ Additionally, Black race was reported as a risk factor for cancer-specific mortality in a recent study of approximately 63 000 patients with DLBCL.^9^ The observed differences in outcomes based on race and ethnicity are further demonstrated by a study of approximately 85 000 patients diagnosed with DLBCL between 1973 and 2011. A higher median overall survival was reported in White patients (5.8 years) compared with non-White races (3.3 years, 2.9 years, and 2.8 years in African American, Asian, and Hispanic patients, respectively).^3^

Historically, clinical trials supporting the approval of therapies for DLBCL have not reported race and ethnicity data or have lacked representation from diverse racial backgrounds. In the United States, 25.2% of the population are a race other than White, and 20.0% are Hispanic or Latino.^10^ Despite this, clinical trials associated with several novel treatments approved in recent years by the US Food and Drug Administration (FDA) for patients with relapsed or refractory (R/R) DLBCL in second-line (2L) treatment and beyond (including the anti-cluster of differentiation 19 [CD19]–directed chimeric antigen receptor T-cell [CAR-T] therapies [axicabtagene ciloleucel, lisocabtagene maraleucel, and tisagenlecleucel], the CD19-directed antibody–drug conjugate loncastuximab tesirine, and the anti-CD19 monoclonal antibody tafasitamab),^11–14^ were primarily conducted in White patients or did not report race and ethnicity data.^15–20^ The L-MIND trial, which was the basis for approval of tafasitamab in combination with lenalidomide for patients with R/R DLBCL, also lacked racial diversity, with 89% of enrolled patients identifying racially as White.^21^^,^^22^ Moreover, in a recent multicenter retrospective study exploring real-world outcomes in patients receiving tafasitamab plus lenalidomide for R/R DLBCL in an academic setting, 90% of patients identified racially as White.^23^

Concerns have been raised by the FDA regarding underrepresentation of minority racial and ethnic groups in clinical studies.^24^ Consequently, the FDA released draft guidance for diversity plans to improve the enrollment of racial and ethnic minority populations in clinical trials, aiming to ensure adequate representation and that study outcomes reflect the populations expected to receive the therapy once approved.^25^ As real-world studies often include larger and more heterogeneous populations than clinical trials, they serve as an important tool for bridging gaps in knowledge about treatment patterns and outcomes among underrepresented groups.

To better understand characteristics, treatment patterns, and outcomes of adult patients who initiated tafasitamab for R/R DLBCL in the real-world setting in the United States, a retrospective, physician-abstracted medical chart review was conducted.^26^ In this analysis, results from the real-world tafasitamab study are presented by racial and ethnic subgroups.

## Methods

### Study design and data source

This retrospective, observational, physician-abstracted medical chart review included patients with R/R DLBCL in the United States who received tafasitamab. Physicians were recruited from Cardinal Health’s Oncology Provider Extended Network (OPEN) of oncologists, hematologists, and urologists across the United States. The study adhered to all standard research guidelines and received approval and exemption for obtaining informed consent from patients by a central institutional review board.

Approximately 1150 OPEN physicians were invited via email, 23 of whom voluntarily participated by completing electronic case report forms on eligible patients; each physician could contribute data for up to 10 randomly selected patients. Physicians were first instructed to compile a complete list of all eligible patient charts in chronological order, beginning with the earliest qualifying case, based on predefined inclusion and exclusion criteria. From this comprehensive list, physicians were instructed to randomly select up to 10 patients for data abstraction. This approach ensured that all eligible patients were identified prior to sampling and minimized the potential for physician-driven selection bias. Physicians provided information regarding their practice characteristics and abstracted retrospective demographic, clinical, treatment, and outcomes-related data from patients’ medical records. Data were collected from February 22, 2023 to March 29, 2023; and subsequently, between December 18, 2023 and January 31, 2024, the physicians were asked to provide additional follow-up data on patients indicated as still alive during initial data collection.

The primary objectives of the overall study were to describe eligible patients’ demographics, clinical characteristics, treatment sequencing, and effectiveness outcomes. Secondary objectives included evaluating the association of baseline demographic and clinical characteristics with real-world effectiveness measures, including progression-free survival (rwPFS) and overall survival (rwOS). In this analysis, we report data by race and ethnicity.

### Study population

Physicians identified eligible patients with a diagnosis of R/R DLBCL who had initiated tafasitamab (with or without concomitant lenalidomide) on or after October 21, 2020, and who were aged ≥18 years at the start of tafasitamab treatment. Patients were required to have at least 4 months of follow-up from the start of tafasitamab treatment; however, those who died during this period were included if otherwise eligible. Patients who received tafasitamab as part of an interventional clinical trial were excluded.

### Statistical analysis

Data from the 2 collection periods were combined to summarize findings using descriptive statistics. In the electronic case report forms, race categories were American Indian or Alaska Native, Asian, Black or African American, Native Hawaiian or Other Pacific Islander, White, and Unknown. Physicians could select more than 1 race from the options provided. During analysis, physician responses were reclassified and results were summarized into mutually exclusive categories by race (White, Black or African American, Other, Unknown) and ethnicity (non-Hispanic, Hispanic, Unknown per the electronic case report forms). The “Other” racial category included patients with mixed race or a race other than White, Black or African American, or unknown race. Effectiveness outcomes are not reported for subgroups with fewer than 20 patients due to small sample size. Unadjusted time‑to‑event endpoints were analyzed using the Kaplan–Meier method.

Effectiveness outcomes included real-world overall response rate (rwORR), duration of response, rwPFS, and rwOS. rwORR was defined as the best response at any point during tafasitamab therapy and was calculated as the sum of patients with reported complete response (CR) and partial response (PR) to tafasitamab divided by all patients within the cohort. Duration of response was measured from the first observed CR or PR to the time of physician-reported disease progression or death from any cause, whichever occurred first. For patients without a documented progression event, a progression event was imputed at the date of discontinuation if the reported reason for discontinuation was disease progression. Patients without a documented progression event were censored at the start date of a new anticancer therapy if discontinuation was not attributed to disease progression. Those without a documented progression event or a new anticancer therapy start date were censored at the date of discontinuation or, if still receiving tafasitamab, at the date of last encounter. rwPFS was the time from tafasitamab initiation to physician-reported disease progression or death from any cause, whichever occurred first. RwOS was defined as time from tafasitamab initiation to death from any cause. Potential associations between patient and treatment characteristics, and rwPFS and rwOS were assessed using univariable analysis; variables with *P *≤ .2 were included in multivariable Cox proportional hazards models. Candidate covariates included demographic and clinical characteristics (eg, age, Eastern Cooperative Oncology Group performance status), and treatment-related factors (eg, line of therapy at tafasitamab initiation), in addition to race and ethnicity. Backward selection with *P *< .05 was performed to identify factors associated with real-world PFS and rwOS in patients who had initiated tafasitamab for R/R DLBCL; race and ethnicity were evaluated as candidate covariates within the selection approach. Akaike information criterion determined best model fit. Data analyses were performed using SAS statistical software, version 9.4 (SAS Institute).

## Results

### Prescriber characteristics

Twenty-three physicians from unique sites across the United States contributed patient data to this study; all 4 geographic regions of the United States were represented (South [39.1%], West [21.7%], Northeast [21.7%], Midwest [17.4%]). Of these physicians, 11 (47.8%) were from urban practices, 9 (39.1%) were from suburban practices, and 3 (13.0%) were from rural practices. Most physicians were from community oncology practice settings (82.6%), and the remaining physicians were from academic medical centers or affiliated teaching hospitals (17.4%). Physicians had a mean (*SD*) of 16.7 (6.3) years in practice. Across all participating physicians, the median (first quartile [Q1] to third quartile [Q3]) number of eligible patients and electronic case report forms submitted per physician was 13 (7-20) and 10 (6-10), respectively, indicating that physicians contributed approximately 75%-80% of their eligible patients. Physicians with ≤10 eligible patients could have contributed all eligible cases.

### Patient demographics and characteristics

A total of 181 eligible patients were included in this analysis. An additional patient met prespecified eligibility criteria but was excluded from the study as they received tafasitamab as bridge therapy to CAR-T, rather than a line of therapy (Figure 1). Of 181 patients, 116 (64.1%) were White, 40 (22.1%) were Black or African American, 14 (7.7%) were of a mixed race or a race other than White, Black or African American, or unknown (Other), and 11 (6.1%) had unknown race; 149 patients (82.3%) were non-Hispanic, 31 (17.1%) were Hispanic, and 1 (0.6%) had unknown ethnicity. At time of initial data collection, 144 patients (79.6%) were still alive, and physicians provided additional follow-up data on 137 of these patients (95.1%).

**Figure 1.**
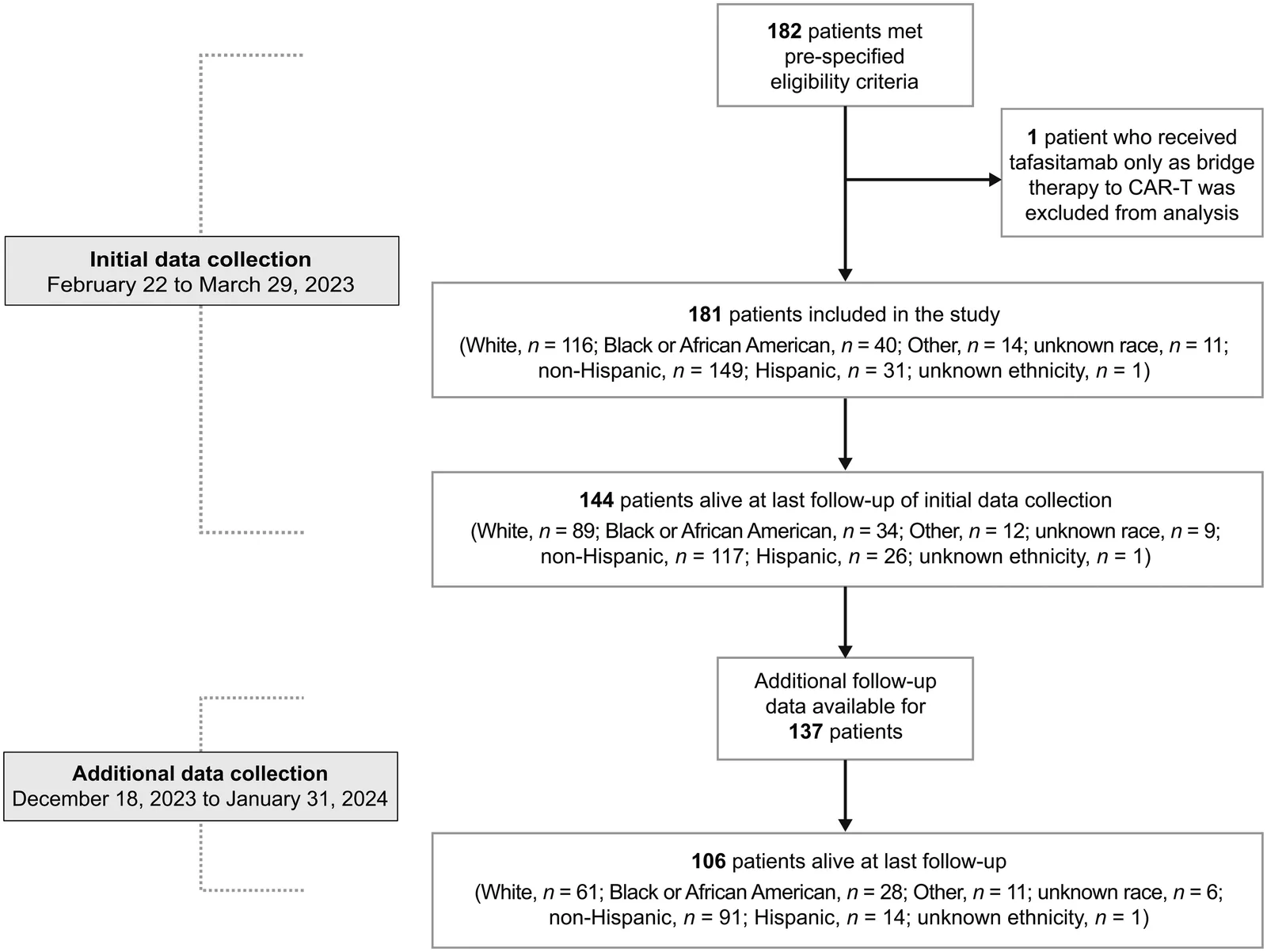
Study design. Abbreviation: CAR-T, chimeric antigen receptor T cell.

Patient characteristics by racial and ethnic subgroups are described in Table 1. Median age (Q1-Q3) at tafasitamab initiation for the overall population was 71.1 (65.0-75.5) years and was similar among each of the subgroups. The proportion of patients with Eastern Cooperative Oncology Group performance status ≥ 2 at time of tafasitamab initiation was highest in the White racial subgroup (52.6%), followed by 42.5% in the Black or African American, and 28.6% in the Other group, and 49.0% in non-Hispanic and 41.9% in Hispanic ethnic subgroups. Among patients with sufficient data available to calculate a revised International Prognostic Index (R-IPI) score (*n *= 169), the majority of patients among all racial and ethnic groups had an R-IPI score of 3-5 at tafasitamab initiation. More than one-fifth of patients from each racial and ethnic subgroup had primary refractory disease, defined as disease progression while receiving first-line (1L) therapy or disease progression occurring ≤6 months after the completion of 1L therapy. Overall, the median (Q1-Q3) follow-up time from initiation of tafasitamab was 14.7 (8.5-17.1) months; across racial and ethnic groups the median (Q1-Q3) follow-up times were 14.5 [8.3-17.8] months for White, 15.3 [10.0-17.0] months for Black or African American, 15.0 [13.8-18.7] months for Other racial groups; and 9.9 [6.0-16.3] months for Hispanic and 14.9 [9.3-17.5] months for non-Hispanic ethnic groups.

**Table 1.** Patient demographic and clinical characteristics by race and ethnicity.

Characteristic	Race^a^			Ethnicity^a^	
	White (*n = *116)	Black or African American (*n = *40)	Other^b^ (*n = *14)	Non-Hispanic (*n = *149)	Hispanic (*n = *31)
**Sex at birth, male, *n* (%)**	65 (56.0)	25 (62.5)	6 (42.9)	88 (59.1)	14 (45.2)
**Age at baseline,^c^ median (Q1-Q3) years**	71.3 (64.9-76.3)	71.2 (66.5-74.6)	70.1 (67.1-75.2)	71.1 (65.2-75.2)	71.9 (62.3-78.1)
**US geographic location, *n* (%)**					
** South**	31 (26.7)	16 (40.0)	1 (7.1)	42 (28.2)	7 (22.6)
** Midwest**	29 (25.0)	7 (17.5)	2 (14.3)	37 (24.8)	1 (3.2)
** West**	29 (25.0)	2 (5.0)	8 (57.1)	33 (22.1)	13 (41.9)
** Northeast**	27 (23.3)	15 (37.5)	3 (21.4)	37 (24.8)	10 (32.3)
**ECOG PS at baseline,^c^ *n* (%)**					
** 0-1**	55 (47.4)	23 (57.5)	10 (71.4)	76 (51.0)	18 (58.1)
** ≥2**	61 (52.6)	17 (42.5)	4 (28.6)	73 (49.0)	13 (41.9)
**Cell of origin, *n* (%)**					
** GCB**	55 (47.4)	20 (50.0)	4 (28.6)	69 (46.3)	12 (38.7)
** Non-GCB/ABC**	24 (20.7)	10 (25.0)	3 (21.4)	30 (20.1)	9 (29.0)
** Unknown**	37 (31.9)	10 (25.0)	7 (50.0)	50 (33.6)	10 (32.3)
**Ann Arbor stage at baseline,^c^ *n* (%)**					
** Stage I**	0	0	0	0	0
** Stage II**	5 (4.3)	1 (2.5)	3 (21.4)	8 (5.4)	1 (3.2)
** Stage III**	32 (27.6)	19 (47.5)	4 (28.6)	49 (32.9)	9 (29.0)
** Stage IV**	79 (68.1)	19 (47.5)	7 (50.0)	91 (61.1)	20 (64.5)
** Unknown**	0	1 (2.5)	0	1 (0.7)	1 (3.2)
**Patients with R-IPI score at baseline,^c^ *n* (%)**	109 (94.0)	37 (92.5)	13 (92.9)	140 (94.0)	28 (90.3)
**R-IPI score at baseline,^c^ *n* (% of patients with available data)**					
** 0 (very good prognosis)**	0	0	0	0	0
** 1-2 (good prognosis)**	19 (17.4)	9 (25.0)	4 (28.6)	29 (20.7)	3 (10.7)
** 3-5 (poor prognosis)**	90 (82.6)	27 (75.0)	10 (71.4)	111 (79.3)	25 (89.3)
**Presence of double- or triple-hit B-cell lymphoma at baseline,^c^ *n* (%)**					
** Yes, double-hit**	12 (10.3)	7 (17.5)	1 (7.1)	15 (10.1)	5 (16.1)
** Yes, triple-hit**	0	1 (2.5)	0	1 (0.7)	1 (3.2)
** Tested and found to be negative**	86 (74.1)	25 (62.5)	11 (78.6)	109 (73.2)	20 (64.5)
** Unknown**	18 (15.5)	7 (17.5)	2 (14.3)	24 (16.1)	5 (16.1)
**Primary refractory,^d^ *n* (%)**	25 (21.6)	10 (25.0)	5 (35.7)	37 (24.8)	10 (32.3)
**Primary refractory/early relapse,^e^ *n* (%)**	60 (51.7)	21 (52.5)	9 (64.3)	79 (53.0)	18 (58.1)

Abbreviations: ABC, activated B cell; ECOG PS, Eastern Cooperative Oncology Group performance status; GCB, germinal center B cell; Q, quartile; R-IPI, revised International Prognostic Index; R/R, relapsed or refractory.

a Eleven patients were reported to be of unknown race and are not included in this table; 1 patient was of unknown ethnicity and is not included in this table.

b Patients who were not White only, Black or African American only, or Unknown (includes Asian, Native Hawaiian/Other Pacific Islander, American Indian or Alaska Native, or mixed race).

c At the time of tafasitamab initiation.

d Defined as disease progression while receiving first-line therapy or disease progression occurring ≤6 months after the completion of first-line therapy.

e Defined as disease progression while receiving first-line therapy or disease progression occurring ≤12 months after the completion of first-line therapy.

### Treatment and utilization patterns

Treatment patterns among patients who received tafasitamab for R/R DLBCL are outlined in Table 2 by racial and ethnic subgroups. Most patients received a rituximab plus cyclophosphamide, doxorubicin, vincristine, prednisone (R-CHOP) regimen (87.8%) in the 1L setting, with 86.2% receiving R-CHOP and 1.7% receiving R-low dose CHOP (R-miniCHOP). Most patients (71.8%) received tafasitamab as 2L therapy for R/R DLBCL; 6.0% of White, 3.4% of non-Hispanic, and 9.7% of Hispanic patients received tafasitamab as fourth-line therapy and beyond. None of the Black or African American patients or patients classified as Other race received tafasitamab beyond the third-line setting. Across all racial and ethnic subgroups, most patients received tafasitamab treatment in combination with lenalidomide (*n *= 174, 96.1%), with lenalidomide 25 mg as the most common starting dose (Table 2). The most frequent lenalidomide dosing schedule was once daily for 21 days, repeated every 28-day cycle (among 97.1% of patients). Overall, the median (95% CI) duration of treatment with tafasitamab was 11.0 (9.4-12.2) months (Table 2). The most common reason for tafasitamab discontinuation across all racial and ethnic subgroups was disease progression (White, 67.8%; Black or African American, 77.8%; Other, 85.7; non-Hispanic, 70.2%; Hispanic, 75.0%).

**Table 2.** Treatment patterns among patients who received tafasitamab for R/R DLBCL by race and ethnicity.^a^

Characteristic	Race^b^			Ethnicity^b^	
	White	Black or African	Other^c^	Non-Hispanic	Hispanic
	(*n = *116)	American (*n = *40)	(*n = *14)	(*n = *149)	(*n = *31)
**Line of therapy in which tafasitamab was received, *n* (%)**					
** 2L**	80 (69.0)	31 (77.5)	12 (85.7)	109 (73.2)	20 (64.5)
** 3L**	29 (25.0)	9 (22.5)	2 (14.3)	35 (23.5)	8 (25.8)
** 4L**	5 (4.3)	0	0	3 (2.0)	2 (6.5)
** 5L**	2 (1.7)	0	0	2 (1.3)	1 (3.2)
**Time from initial diagnosis to initiation of tafasitamab, median (Q1-Q3), months**	21.0 (13.2-37.0)	23.3 (12.6-34.8)	18.0 (15.0-21.4)	20.0 (12.4-35.0)	19.0 (14.0-34.0)
**Received ASCT before tafasitamab, *n* (%)**	17 (14.7)	2 (5.0)	1 (7.1)	17 (11.4)	4 (12.9)
**Received CAR-T therapy before tafasitamab, *n* (%)**	4 (3.4)	1 (2.5)	0	4 (2.7)	2 (6.5)
**Subsequent CAR-T therapy, *n* (%)**	6 (5.2)	0	3 (21.4)	8 (5.4)	1 (3.2)
**Received tafasitamab in combination with lenalidomide, *n* (%)**	109 (94.0)	40 (100.0)	14 (100.0)	143 (96.0)	31 (100.0)
**Lenalidomide starting dose, *n* (%)**					
** 25 mg**	45 (38.8)	13 (32.5)	6 (42.9)	55 (36.9)	13 (41.9)
** 20 mg**	29 (25.0)	9 (22.5)	4 (28.6)	34 (22.8)	10 (32.3)
** 15 mg**	19 (16.4)	7 (17.5)	1 (7.1)	24 (16.1)	5 (16.1)
** 10 mg**	16 (13.8)	10 (25.0)	3 (21.4)	29 (19.5)	2 (6.5)
** 5 mg**	0	1 (2.5)	0	1 (0.7)	1 (3.2)
** No lenalidomide**	7 (6.0)	0	0	6 (4.0)	0
**Tafasitamab discontinued at most recent follow-up, *n* (%)**	87 (75.0)	27 (67.5)	7 (50.0)	104 (69.8)	24 (77.4)
**Tafasitamab TTD^d^, months, median (95% CI)**	10.6 (8.8-12.0)	11.8 (9.1-15.7)	14.1 (6.1-NE)	11.2 (9.8-12.4)	8.8 (5.6-12.8)

Abbreviations: 2L, second-line; 3L, third-line; 4L, fourth-line; 5L, fifth-line; ASCT, autologous stem cell transplantation; CAR-T, chimeric antigen receptor T-cell; DLBCL, diffuse large B-cell lymphoma; NE, not evaluable; Q1, first quartile; Q3, third quartile; R/R, relapsed or refractory, TTD, time to treatment discontinuation.

a The end of a line of therapy was determined as the discontinuation of a treatment regimen. If a drug was initiated alongside ongoing treatment, this was considered a new line of therapy; however, bridge therapy followed by CAR-T therapy and salvage chemotherapy followed by ASCT were considered single lines of therapy.

b Eleven patients were reported to be of unknown race and are not included in this table; 1 patient was of unknown ethnicity and is not included in this table.

c Patients who were not White only, Black or African American only, or Unknown (includes Asian, Native Hawaiian or Other Pacific Islander, American Indian or Alaska Native, or mixed race).

d Difference between date of discontinuation for any reason and date of initiation; patients still on therapy at data collection were censored at last follow-up date.

A total of 21 (11.6%) patients overall received autologous stem cell transplant (ASCT) as 2L treatment prior to tafasitamab; among these patients, 17 were White, 2 were Black or African American, 1 was classified as Other, and 1 had unknown race; 17 were non-Hispanic and 4 were Hispanic. Six (3.3%) patients overall received CAR-T therapy prior to tafasitamab. At the time of most recent data abstraction, 106 patients (58.6%) were alive, 53 (50.0%) of whom were still receiving tafasitamab.

### Effectiveness outcomes

Of the 181 patients, 170 (93.9%) had disease response data available. Disease response data availability and the criteria used to determine disease response by racial and ethnic subgroups are summarized in Table 3. The rwORR (95% CI) across racial and ethnic groups was 73.3% (65.2-81.3) for White patients, and 82.5% (70.7-94.3) for Black or African American patients; and 74.5% (67.5-81.5) for non-Hispanic patients and 67.7% (51.3-84.2) for Hispanic patients (Figure 2). Additional unadjusted outcomes measures among racial and ethnic subgroups are presented in Table 4.

**Figure 2.**
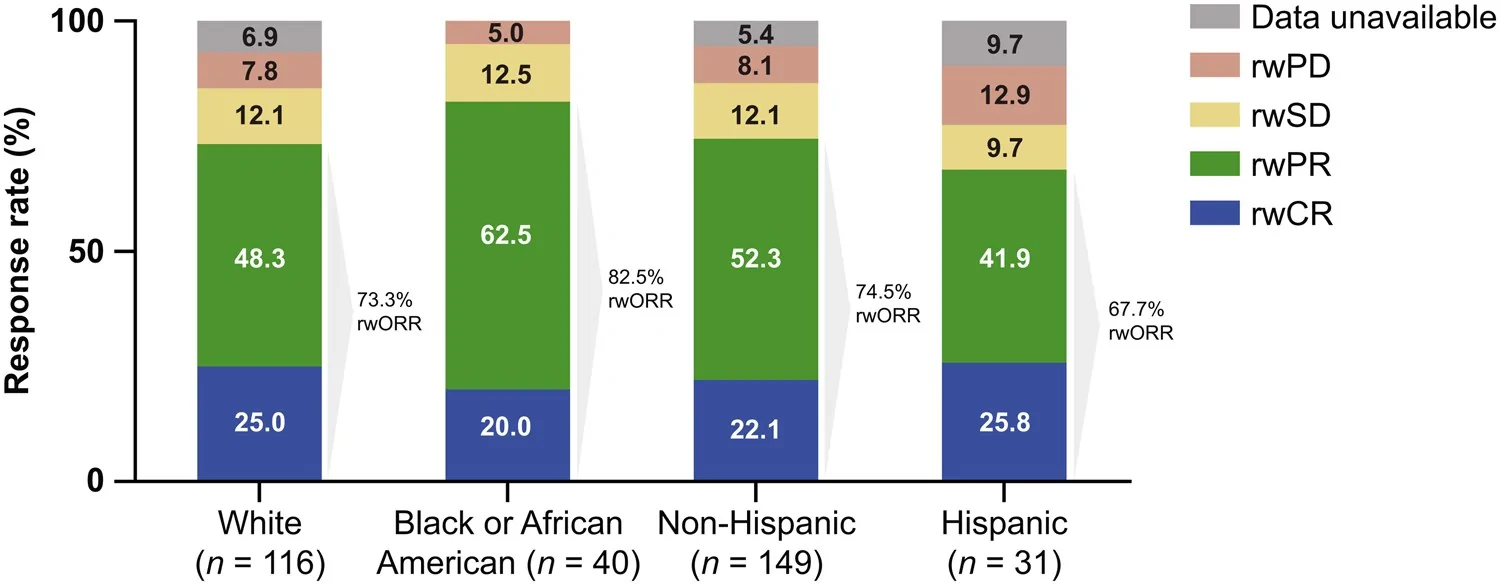
Real-world best responses for patients who received tafasitamab by race^a^ and ethnicity. Abbreviations: rwCR, real-world complete response; rwORR, real-world overall response rate; rwPD, real-world progressive disease; rwPR, real-world partial response; rwSD, real-world stable disease. ^a^Real-world best response not presented for the Other racial group because the number of patients is fewer than 20.

**Table 3.** Number of patients with available disease response data and criteria used to assess response.

Variable	Race^a^^,^^b^		Ethnicity^a^	
	White	Black or African	Non-Hispanic	Hispanic
	(*n = *116)	American (*n = *40)	(*n = *149)	(*n = *31)
**Patients with disease response available, *n* (%)**	108 (93.1)	40 (100.0)	141 (94.6)	28 (90.3)
**Response criteria utilized for assessing response, *n* (%, among those with response data available)**				
** Cheson 2007**	29 (26.9)	11 (27.5)	40 (28.4)	5 (17.9)
** Lugano 2014**	79 (73.1)	29 (72.5)	101 (71.6)	22 (78.6)
** Other**	0 (0.0)	0 (0.0)	0 (0.0)	1 (3.6)

a Eleven patients were reported to be of unknown race and are not included in this table; 1 patient was of unknown ethnicity and is not included in this table.

b Real-world best response not presented for the Other racial group because the number of patients is fewer than 20.

**Table 4.** Unadjusted treatment outcomes by race and ethnicity.

Effectiveness measures	Race^a^^,^^b^		Ethnicity^a^	
	White (*n = *116)	Black or African American (*n = *40)	Non-Hispanic (*n = *149)	Hispanic (*n = *31)
**rwPFS,^c^** **median (95% CI), months**	11.0 (9.2-12.8)	13.6 (9.2-15.8)	11.9 (10.1-15.3)	9.2 (5.6-11.7)
**rwOS,^d^** **median (95% CI), months**	19.2 (14.2-NE)	NR (NE-NE)	NR (18.3-NE)	14.7 (7.3-NE)
**rwDOR,^e^** **median (95% CI), months**	8.7 (7.8-15.2)	9.6 (5.4-13.3)	10.0 (8.5-15.2)	6.7 (3.6-8.8)
**rwORR^f^** **(95% CI), %**	73.3 (65.2-81.3)	82.5 (70.7-94.3)	74.5 (67.5-81.5)	67.7 (51.3-84.2)

Abbreviations: CR, complete response; DOR, duration of response; NE, not estimable; NR, not reached; ORR, overall response rate; OS, overall survival; PFS, progression-free survival; PR, partial response; rw, real-world.

a Eleven patients were reported to be of unknown race and are not included in this table; 1 patient was of unknown ethnicity and is not included in this table.

b Real-world best response not presented for the Other racial group because the number of patients is fewer than 20.

c rwPFS was defined as the time from tafasitamab initiation to disease progression or death from any cause, whichever occurred first.

d Time from tafasitamab initiation to death from any cause.

e Among patients for whom a CR or PR is observed during tafasitamab treatment, DOR was calculated as the time from when CR or PR was first observed to the time of physician-reported disease progression or death from any cause, whichever occurred first.

f Sum of patients with reported rwCR and patients with real-world partial response (rwPR) as their reported best response to tafasitamab divided by the total number of patients in the cohort and reported as a percent.

Multivariable analyses were conducted to evaluate factors associated with rwPFS and rwOS; however, as the primary objective of this analysis was to assess outcomes by race and ethnicity, detailed results for non–race/ethnicity covariates are not shown, but can be found in the primary report describing results from the overall study population.^26^ In multivariable Cox proportional hazards-based analyses, neither race nor ethnicity was found to be significantly associated with rwPFS or rwOS. Univariable analyses identified Hispanic ethnicity as potentially associated with increased risk of disease progression (vs non-Hispanic/unknown; hazard ratio [HR] 1.6, 95% CI, 1.0-2.5; *P *= .049) and mortality (HR 1.7, 95% CI, 1.0-3.0; *P *= .046); however, this was not a significant variable when adjusted for other patient and treatment characteristics in the final multivariable Cox models. In univariable analyses, Black or African American race was not found to be associated with increased risk of progression (vs White; HR 0.88, 95% CI, 0.6-1.4; *P *= .564). Based on the *P* value of Black or African American race (vs White; HR 0.58, 95% CI 0.31-1.08; *P *= .09) in univariable analysis of overall survival, this variable was included in the multivariable Cox model; however, it was not found to be significantly associated with increased risk of mortality when adjusted for other factors in the full model.

## Discussion

In this retrospective medical chart review study, we evaluated the characteristics, treatment patterns, and outcomes by race and ethnicity of 181 patients who received tafasitamab for R/R DLBCL in the real-world setting. This racially and ethnically diverse study population is reflective of the heterogenous population of patients with R/R DLBCL in the United States, providing valuable real-world insights into the outcomes of tafasitamab among populations that are often underrepresented in clinical trials. Furthermore, patients included in this analysis were predominantly treated at community-based practices, where most cancer care is delivered to patients in the United States.

Historically, there has been a lack of diversity in clinical studies and subsequent reporting of enrollment data by race and ethnicity. A study of over 20 000 United States-based clinical trials (not limited to oncology) found that 79.7% of patients enrolled in any study during the 20-year analysis period were White, 10.0% Black, 6.0% Hispanic or Latino, and 1.0% Asian, which highlights the underrepresentation of minority race and ethnic groups in clinical trials.^27^ Furthermore, only 43% of these clinical trials reported race or ethnicity data.^27^ The insufficient representation of racial and ethnic minorities in clinical studies results in skewed clinical data and limited understanding of how these groups respond to evidence-based treatments. Reasons for this underrepresentation are considered to be related to mistrust, cost, and lack of information and access to trials; however, when given the opportunity to participate in a clinical trial, patients from minority racial and ethnic groups are as likely to participate as White patients.^28^

Several methods can be implemented to increase the representation of racial and ethnic minorities in clinical studies, including patient coaching, culturally tailored patient information, training for recruiting physicians, and relationship building.^29^ Diverse participation in clinical trials is actively encouraged by the FDA in order to bridge the knowledge gap and identify both intrinsic and extrinsic population-specific factors that may lead to observed differences in pharmacokinetics, efficacy, and safety of treatments in underrepresented groups.^30^ In the absence of diversity in clinical studies, real-world data from diverse populations such as those summarized in this report could support data from clinical trials that may not be as representative of the population.

This study reports race and ethnicity data from a more diverse population in a real-world setting (Black or African American [22.1%], Other [7.7%]; and Hispanic [17.1%]), which supports the value of real-world studies in increasing knowledge and understanding of treatment outcomes for patients from minority racial and ethnic backgrounds. In this study, relatively high rwORRs were observed by physicians across all racial and ethnic groups (73.3% in White patients, 82.5% in Black or African American patients; and 74.5% in non-Hispanic patients and 67.7% in Hispanic patients), highlighting the clinical benefit of tafasitamab treatment in a diverse population. In addition, based on multivariable Cox modeling analysis, neither race nor ethnicity was found to be significantly associated with rwPFS or rwOS. In the phase 2L-MIND study, which was the basis for the accelerated approval by the FDA of tafasitamab in combination with lenalidomide for adult patients with R/R DLBCL who are ineligible for ASCT, the ORR was 60% (95% CI, 48-71)^22^ in the primary analysis and 57.5% (95% CI, 46-69) after long-term follow-up.^31^ As reported previously,^26^ it is also worth noting that the ORR among the overall study population was higher in this study compared to another published real-world study (73% vs 31%) by Qualls et al.^23^ This difference in effectiveness outcome may be partially due to the differences in baseline characteristics and line of therapy in which tafasitamab was received, potentially reflecting variations in clinical practice between community and academic centers.^26^

This analysis, like other real-world studies, may be limited by unobserved data and missing data bias, for example, loss to follow-up or missing events that occurred outside of a clinical setting. Although physicians were not blinded to the study objectives, several design elements were implemented to mitigate selection bias, including predefined eligibility criteria, complete enumeration of eligible patients prior to sampling, and random selection. Moreover, physicians contributed approximately 75%-80% of eligible patients, and many contributed all eligible cases, suggesting that the impact of physician-level selection bias is likely limited. Nevertheless, residual selection bias cannot be entirely excluded, particularly among physicians with a high volume of eligible patients. An additional limitation pertains to the censoring approach used for patients who discontinued tafasitamab without documented disease progression or initiation of a subsequent anticancer therapy. Some of these patients may have remained progression-free after tafasitamab discontinuation; however, disease status after discontinuation was not collected in this retrospective chart review. Consequently, these patients were censored at tafasitamab discontinuation, which may affect the interpretation of time-to-event endpoints. Because disease status following discontinuation was not collected, some patients may have remained progression-free after discontinuation. As a result, progression-free outcomes may have been underestimated in some patients. It is also important to note that treatment patterns observed in this study may not reflect practices of all oncologists managing patients with DLBCL and that source documents were not verified in our study; however, all physicians were required to submit data validation checks, and data that were unable to be validated were excluded. Our study did not collect safety outcomes, which are important for assessing therapeutic effects in diverse populations; we acknowledge the importance of addressing these questions in future studies. Finally, study findings may be impacted by a lack of uniform assessment criteria for some variables including disease response.

In summary, results from our real-world study suggest that the observed effectiveness outcomes among patients receiving tafasitamab for R/R DLBCL were consistent across racial and ethnic subgroups. Multivariable analyses did not identify race or ethnicity as significant predictors of rwPFS or rwOS; however, given the limitations of the study including small sample sizes within racial and ethnic subgroups, further research is needed to confirm these findings.

## Data Availability

De-identified data are not available for this retrospective real-world study. For other data**-**related queries, please contact the corresponding author, Kim Saverno (ksaverno@incyte.com).
